# Late-stage diagnosis and its impact on survival in Pakistani women with cervical cancer: Findings from an institutional cancer registry

**DOI:** 10.12669/pjms.41.10.12176

**Published:** 2025-10

**Authors:** Uzma Shamsi, Sarah Saleem, Namra Usman, Iqbal Azam, Aliya Aziz

**Affiliations:** 1 Uzma Shamsi, MBBS, MSc, PhD. Department of Community Health Sciences (CHS), Aga Khan University Hospital (AKUH), Stadium Road, Karachi, Pakistan; 2 Sarah Saleem, MBBS, FCPS. Department of Community Health Sciences (CHS), Aga Khan University Hospital (AKUH), Stadium Road, Karachi, Pakistan; 3 Namra Usman, MBBS. Department of Community Health Sciences (CHS), Aga Khan University Hospital (AKUH), Stadium Road, Karachi, Pakistan; 4 Iqbal Azam, MSc. Department of Community Health Sciences (CHS), Aga Khan University Hospital (AKUH), Stadium Road, Karachi, Pakistan; 5 Aliya Aziz, MBBS, FCPS. Department of Obstetrics and Gynecology, Aga Khan University Hospital (AKUH), Stadium Road, Karachi, Pakistan

**Keywords:** Cervical cancer, survival, late-stage at diagnosis

## Abstract

**Objective:**

This study aimed to analyze mean age, clinicopathological characteristics, and survival rates of cervical cancer patients in Karachi, Pakistan.

**Methodology:**

A retrospective cohort study was conducted using data from the Cancer Registry at Aga Khan University Hospital (AKUH) between January 1, 2010 to December 31, 2020. Kaplan-Meier estimates were used to calculate one-, three-, and five-year survival rates. Cox proportional hazards regression was employed to identify factors associated with survival. The analysis included 310 women diagnosed with primary cervical cancer between 2010 and 2020.

**Results:**

The mean age at diagnosis was 51.3 years (SD ± 11.3), with most women (83.9%) diagnosed at an advanced stage. Kaplan-Meier survival analysis revealed an overall mean survival time of 176.3 months (SD 18.1), with one-, three-, and five- years survival rates of 86.6%, 78.5%, and 54.4%, respectively. Cox proportional hazards regression identified late-stage diagnosis (HR = 3.64, 95% CI: 1.25-10.59) and smoking history (HR = 3.37, 95% CI: 1.27-8.97) as independent predictors of poorer survival.

**Conclusion:**

The high frequency of late-stage disease and its impact on poor survival in our study highlight the critical challenge and the urgent need for targeted public health interventions. Strategies such as enhanced community awareness campaigns, the implementation of HPV vaccination, improved cervical cancer screening programs, and the promotion of timely medical consultations are essential to facilitate earlier diagnosis and improve survival outcomes.

***List of abbreviations:*** Aga Khan University Hospital (AKUH), adjusted hazard ratios (aHR), confidence intervals (CI), Standard Deviation (SD), Interquartile Range (IQR), Hazard Ratio (HR), Human Papillomavirus (HPV), age standardized incidence rate (ASIR), World Health Organization (WHO), Lower Middle-Income Countries (LMICs), Squamous cell carcinoma (SCC), American Joint Committee on Cancer (AJCC).

## INTRODUCTION

Cervical cancer is the fourth most common cancer in women globally, with an estimated 604,000 new cases and 342,000 deaths in 2020.^1^ The global age-standardized incidence rate (ASIR) is 13.3 per 100,000 woman-years, varying widely (3 to 73 per 100,000) due to differences in screening, HPV vaccination, and sexual behaviors affecting HPV transmission. The WHO Cervical Cancer Elimination Initiative 2020 aims to reduce the ASIR to below 4 per 100,000.^2^ The burden of cervical cancer is much higher in developing regions, accounting for nearly 85% of global incidence and mortality.^3^ The estimated cervical cancer burden in Pakistan is also higher than the WHO target (adjusted ASIR of 7.60, (95% UI 5.98, 10.01)).^3^

Cervical cancer diagnosis occurs across a broad age range, from the 20s to the 60s, with a peak incidence in women aged 35-44 years. The median age at diagnosis is approximately 50 years, and around 20% of cases are in women over 65, according to the National Cancer Institute.^4^ Squamous cell carcinoma is the most prevalent type of cervical cancer, while adenocarcinoma of cervix has a worse prognosis.^5^ In a study in India, old age and late-stage at the time of diagnosis were the determinants of poor survival.^6^ Another study in Japan also reported that the older age at diagnosis was associated with lower survival rates.^7^ A study among French population showed that elderly women diagnosed with cervical cancer at an advanced stage and not receiving standard care had an elevated risk of mortality.^8^ A study in eastern China showed that timely detection and screening programs there have resulted in a 90.9% 5-year relative survival rate for cervical cancer patients.^9^ A stud conducted in Pakistan reported high prevalence of HPV in precancerous lesions of cervix.^10^

Despite progress in cervical cancer prevention, screening and treatment, a significant burden of advanced cervical cancer and high mortality persist among Pakistani women. There is a dearth of information regarding its survival and factors affecting it. The objective of this study was to assess survival rates among affected women, and evaluate factors influencing survival.

## METHODOLOGY

This study was a retrospective cohort study, utilizing a hospital-based cancer registry database, a comprehensive cancer registry of AKUH, Karachi, Pakistan. This database maintains data from patients visiting AKUH from all over Pakistan and has the potential to guide national cancer control and prevention policy in Pakistan. The data extracted included sociodemographic factors and source of payment for the treatment. Disease was classified according to the International Classification of Diseases for Oncology, *3^rd^ edition (*ICD-O-3): CIN1 (D06.0, CIN1: D06.0, CIN2: D06.1, CIN3: D06.1 (Squamous cell carcinoma of cervix: In situ: D06.0, Invasive: C53.0), Adenocarcinoma of cervix (In situ: D06.7, Invasive: C53.1), Adeno-squamous carcinoma of cervix: C53.9, Small cell carcinoma of cervix: C53. Tumor staging followed the AJCC 8th edition. Other variables included treatment modalities with dates, surgery, chemotherapy, radiation, last follow-up, and vital status at the time of last follow-up (alive or deceased). The study included all female patients diagnosed with biopsy-proven primary cervical cancer at AKUH between January 1, 2010, and December 31, 2020.

### Ethics approval and consent to participate:

ERC exemption was sought from the Ethical Review Committee ERC AKUH (ERC reference # 8857). Dated October 09, 2023.

### Statistical analysis:

Overall survival (OS) was calculated in months from the date of diagnosis to the date of death or the date of last follow-up (censored). One-, three-, and five-year OS rates were estimated using the Kaplan-Meier method. Cox proportional hazards regression model was conducted to determine independent predictors of OS. All statistical analyses were conducted using IBM SPSS Statistics version 25 (IBM Corp., Armonk, NY, USA).

## RESULTS

The baseline sociodemographic characteristics of the 310 women with cervical cancer and results of univariate analyses are shown in Table-I. Tobacco use was associated with poorer survival (HR = 3.57, 95% CI: 1.36, 9.37).

**Table-I:** Univariate Analysis of Sociodemographic and Clinical Factors Associated with Cervical Cancer Survival in Karachi, Pakistan (n=310).

Characteristics	n	%	Crude HR* (95% CI)
***Age at diagnosis (years)***			
>60	70	22.6	1.10(0.40, 3.08)
40-60	190	61.3	0.51(0.21, 1.27)
22-39	50	16.1	Ref
***Marital Status***			
Married	281	90.6	0.52(0.18, 1.48)
Single/widow/separated	29	9.4	Ref
***Occupation***			
Housewife	189	61	1.55(0.36, 6.65)
Employed	26	8.4	Ref
***Province***			
Sindh	250	80.6	2.76(0.66, 11.66)
Outside Sindh	60	19.4	Ref
***Family history of cervical cancer***			
Yes	7	2.3	0.97(0.13, 7.24)
No/unknown	303	97.7	Ref
***Family history of any cancer***			
Yes	44	14.2	1.13(0.46, 2.77)
No/unknown	266	85.8	Ref
***Tobacco use***			
Yes	17	5.5	3.57 (1.36, 9.37)
No	293	94.5	Ref
***Payment source***			
Uninsured (self-payment)	301	97.1	0.62(0.08, 4.60)
Insured/financial assistance	9	2.9	Ref

Late-stage disease was associated with significantly poorer survival compared to early-stage disease (HR = 3.83, 95% CI: 1.32, 11.13) as shown in Table-II. Similarly, patients who did not undergo surgery had poorer survival than those who did (HR = 2.23, 95% CI: 1.07, 4.65). Chemotherapy was associated with improved survival (HR = 0.13, 95% CI: 0.02, 0.95). Patients receiving standard-of-care (SOC) treatment, including surgery, radiotherapy, and chemotherapy, showed improved survival (HR = 0.24, 95% CI: 0.07, 0.83).

The results of the multivariable Cox regression analysis of predictors of survival in cervical cancer patients in Karachi, Pakistan are presented in Table-III.. After adjusting for potential confounders, late-stage disease remained significantly associated with poorer survival (adjusted HR = 3.64, 95% CI: 1.25, 10.59). Tobacco use also remained an independent predictor of poorer survival (adjusted HR = 3.37, 95% CI: 1.27, 8.97).

**Table-II:** Univariate analysis of tumor characteristics associated with cervical cancer survival in Karachi, Pakistan (n=310).

Tumor site - Primary	n	%	Crude HR* (95%CI)
Endocervix	13	4.2	1.64(0.39, 6.89)
Cervix uterine	297	95.8	Ref
***Histology of tumor***			
Squamous cell carcinoma (SCC)	232	74.8	0.75(0.30, 1.87)
Adenocarcinoma	32	10.3	1.04(0.29, 3.75)
Adeno-squamous /end/clear cell	46	14.8	Ref
***Grade***			
Well differentiated	13	4.2	0.93(0.11, 7.75)
Moderately differentiated	143	46.1	1.07(0.41, 2.80)
Poorly differentiated	61	19.7	Ref
***AJCC Stage***			
Late -stage (2, 2B, 3&4)	184	59.4	3.83(1.32, 11.13)
Early Stage (0, 1A, 1B, 2A)	101	32.6	Ref
***Recurrence***			
Yes	78	25.5	0.48(0.23, 1.04)
No	144	47.1	0.01(0.00, 0.11)
Never disease free	84	27.5	Ref
***Surgery***			
Surgery not done	145	46.8	2.23(1.07, 4.65)
Surgery done	165	53.2	Ref
***Type of surgery***			
Radical hysterectomy	44	31.9	0.25(0.03, 2.00)
Simple hysterectomy	94	68.1	Ref
***Surgery hospital***			
AKUH	78	25.2	0.47(0.19, 1.12)
Outside AKUH	67	21.6	0.43(0.16, 1.15)
Not done	165	53.2	Ref
***Radiation (EBRT)***			
Yes	231	74.5	1.24(0.51, 3.04)
No	79	25.5	Ref
***Chemotherapy***			
Yes	218	70.3	0.13(0.02, 0.95)
No	92	29.7	Ref
***Brachytherapy & EBRT***			
Yes	160	51.6	0.56(0.27, 1.15)
No	150	48.4	Ref
***Surgery treatment modalities***			
Surgery only	60	19.4	0.14(0.03,0.66)
Chemoradiotherapy (CRT)	116	37.4	0.41(0.13,1.26)
Standard-of-Care (SOC) (Surgery + Radiotherapy + Chemotherapy)	85	27.4	0.24 (0.07, 0.83)
None	49	15.8	Ref

**Table-III:** Multivariable Cox regression analysis of factors associated with cervical cancer survival in Karachi, Pakistan (n=310).

Variables	Adjusted HR* (95% CI)
***AJCC stage***	
Late-stage (2 B, 3&4)	3.64(1.25, 10.59)
Early Stage (0, 1A, 1B &2A)	Ref
***Tobacco use***	
Yes	3.37(1.27, 8.97)
No	Ref

Adjusted for sociodemographic factors, tumor characteristics and treatment modalities.

## DISCUSSION

Our study has reported that the mean age of Pakistani women at the time of cervical cancer diagnosis was 51.3 years (SD ± 11.3). The results showed an association between AJCC late-stage and poor survival in cervical cancer. Our study findings are consistent with a few other studies conducted in Japan, Kenya, and California.^11^-^13^ Additionally, a study from the Netherlands reported that patients who had advanced-stage cervical cancer with extensive nodal involvement, showed higher mortality (HR=1.63; 95% CI= 1.19, 2.24).^14^ Similarly, a study done in Denmark also found an association between higher survival rate early-stage disease in cervical cancer.^15^,^16^

**Fig.1:**
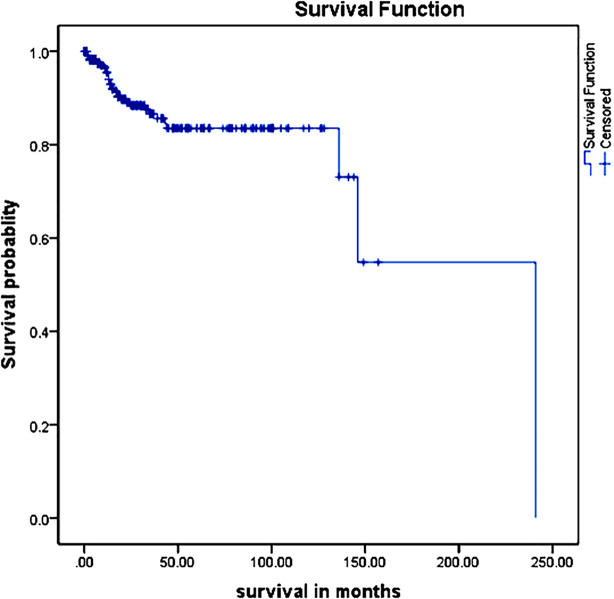
Kaplan-Meier Curve Showing Overall Survival of Cervical Cancer Patients in Karachi, Pakistan.

Tobacco use was a significant predictor of poor survival. Similarly, a study conducted in Sacramento, California, showed that past and current smoking were associated with poor survival including both disease free and overall survival outcomes.^16^ Furthermore, a population-based survival analysis of 2661 women also reported that current smoking was associated with reduced cervical cancer survival (aHR=1.21; 95% CI=1.01-1.46).^17^ Likewise, a study based on global data indicated a correlation between tobacco use with cervical cancer incidence and its association with poor prognosis.^18^ Additionally, research conducted in Washington, USA, highlighted the detrimental impact of tobacco use on survival among women infected with HPV 18 or 45 cervical carcinoma.^19^

This study has demonstrated an overall survival rate for cervical cancer of 54.4%, with one-year survival at 95.0%, and three-years at 86.6%. Our survival rates are similar to those reported in India 20,21 Similarly, a study conducted in Malaysia showed survival rates at one, and three which were 94.1%, and 79.3% respectively.^22^ However, a study in Ghana, East Africa showed lower overall survival rates at one-, and three-years were 76.5%, and 51.5%, respectively.^23^ Analysis of global trend in the survival rates in a study in Indonesia, reported the lower overall survival rates of cervical cancer up to the third year at 76%, 65%, and 59% respectively.^24^

A study conducted in the United States reported a marginal increase in the three-year relative survival rate of cervical cancer patients, increasing from 73.1% to 73.5%, between 2007 and 2011. Due to better screening and treatment, it is anticipated that the three-year survival rate will reach 74.3% between 2017 and 2021.^25^ These variations in survival rates emphasize the disparities in cervical cancer screening, healthcare access, quality of treatment, and early detection practices across different regions. Addressing these challenges is crucial for improving the accuracy and comprehensiveness of survival rate data and for implementing effective interventions to enhance patient outcomes.

### Strengths:

A major strength of this study is that it is among the first to ascertain factors associated with survival rates in Pakistani women. Another major strength of this study is the high quality of local data from the cancer registry at AKUH, and standardized data reporting with a comprehensive set of variables using CNext software for quality data.

### Limitations:

Nonetheless, our study has a few limitations, such as the use of a single-center hospital-based cancer registry, due to lack of a nationwide population-based cancer registry. However, AKUH serves as a referral center, attracting patients from all over Pakistan, thus providing a diverse and representative sample for the study. Another limitation is the lack of specific treatment information for patients who were lost to follow-up, often due to financial barriers at this private hospital. Therefore, larger prospective and multicenter studies are crucial to identify definitive prognostic factors for cervical cancer outcomes. Nonetheless, our findings provide a baseline for future research and interventions aimed at reducing the burden of late-stage cervical cancer in Pakistan.

## CONCLUSION

The findings of this study underscore the significant impact of late-stage diagnosis on survival, highlighting the urgent need for improved early detection and intervention strategies. This further supports the implementation of national HPV vaccination program as a primary preventive measure and targeted public health initiatives are needed.

## Data Availability

the dataset used and analyzed during the current study is available on reasonable request from the principal investigator, Dr. Uzma Shamsi, email: uzma.shamsi@aku.edu. **US, SS:** Literature search, Conception and design **US, IA, SS, NU:** Data collection, data analysis, and interpretation **US, SS, AA, NU:** Critical Review, Manuscript writing. All authors Final approval of manuscript and accountable for all aspects of the work.
